# Cone-Beam Computed Tomography (CBCT)-Based Online Adaptive Radiation Therapy (oART) for a Prostate Cancer Patient With Inflammatory Bowel Disease and Bilateral Total Hip Arthroplasty: A Case Report

**DOI:** 10.7759/cureus.68990

**Published:** 2024-09-09

**Authors:** Matthew Webster, Olga M Dona Lemus, Sean Tanny, Michael Cummings, Dandan Zheng

**Affiliations:** 1 Radiation Oncology, University of Rochester, Rochester, USA

**Keywords:** adaptive radiotherapy, cone-beam computed tomography (cbct), inflammatory bowel disease, metal artifacts, prostate cancer

## Abstract

This case report addresses the complex management of a patient with concurrent prostate cancer, inflammatory bowel disease (IBD), and bilateral total hip arthroplasty, and demonstrates the efficacy of cone-beam computed tomography (CBCT)-guided daily online adaptive radiation therapy (oART) and advanced imaging techniques in overcoming significant treatment challenges.

A 68-year-old male with a history of ulcerative colitis and bilateral hip prostheses was diagnosed with high-risk prostate cancer. Conventional radiation therapy modalities, including external beam radiation therapy (EBRT), proton therapy, and magnetic resonance imaging (MRI)-based oART, faced limitations because of the patient’s comorbidities and metallic implants. Daily oART, using the Ethos platform (Varian Medical Systems, Palo Alto, CA, USA) with HyperSight™ metal artifact reduction (MAR) imaging, was employed to enhance treatment efficacy.

The daily oART treatment on the Ethos platform facilitated the successful delivery of a therapeutic dose while sparing healthy tissues, and the treatment was successful without an IBD flare-up. Daily oART also optimized the target dose while best sparing the critical organs based on the patient’s daily anatomy. The HyperSight MAR algorithm significantly reduced imaging artifacts caused by the hip prostheses, enabling accurate identification of the prostate, bladder, and surrounding organs. The oART workflow was delivered without technical challenges, with a total session time of 20 to 30 minutes, similar to our typical prostate patients without hip implants. Despite the complex anatomy and comorbid conditions, the treatment plan met all organ-at-risk constraints and delivered the prescribed dose to the target volumes.

Ethos oART with HyperSight provided an effective solution for treating a patient with concurrent prostate cancer, IBD, and bilateral hip arthroplasty. The patient’s case was successfully treated without complications, despite such challenging clinical and technical scenarios.

## Introduction

This case report presents a particularly challenging patient with concurrent prostate cancer with nodal involvement, inflammatory bowel disease (IBD), and bilateral total hip arthroplasty. Both IBD and prostheses individually pose significant challenges in managing prostate cancer with radiation therapy. When combined, most treatment techniques, including traditional external beam radiation therapy (EBRT), proton therapy, and magnetic resonance imaging (MRI)-based online adaptive radiation therapy (oART), face limitations in treating such a complex patient. However, as presented here, the use of daily oART and newly developed HyperSight™ metal artifact reduction (MAR) imaging, using the Ethos platform (Varian Medical Systems, Palo Alto, CA, USA), allows for an otherwise unachievable combination of imaging and treatment quality.

The use of radiation therapy to treat prostate cancer has been linked to gastrointestinal (GI) and genitourinary side effects [1-3]. One of the largest challenges for all patients undergoing radiation therapy for prostate cancer is delivering a therapeutic dose to the treatment target while maintaining as low a dose to the surrounding healthy tissue and organs as is achievable. For most patients, the toxicities in these regions are considered unpleasant but manageable. However, for patients with preexisting comorbidities such as ulcerative colitis and Crohn’s disease, the GI toxicities can be severe enough to contraindicate radiation therapy [4-6]. Thus, it is essential to control the radiation dose to the bowel in these patients to reduce the risk of acute IBD flare-ups and higher-grade toxicities. The location and proximity of the bowel relative to the radiation target necessitate high precision and accuracy in contour delineation, as well as in radiation delivery. This is further complicated because the size, shape, and location of the target and bowel can change daily due to organ peristalsis and regular bowel, bladder, and rectal filling. To avoid delivering an unnecessary radiation dose to the bowel, oART allows for the creation of a new plan each day, based on the original, to be delivered [7]. There are several platforms currently available for this, most notably Ethos [8,9], and two MRI-based oART machines, MRIdian (ViewRay, Mountain View, CA, USA) and Unity (Elekta AB, Stockholm, Sweden) [10-12].

The treatment of the patient presented here was further complicated by bilateral total hip arthroplasty. The high-Z material in prostheses is known to cause significant artifacts in 3D imaging [13]. A single high-Z prosthesis can cause artifacts that present as bright and dark streaking. When multiple high-Z prostheses are present in the same axial plane, the region between them can become almost completely dark, with almost no useful anatomical information. These streak artifacts can partially or fully obscure the bladder and prostate gland. This decreases the sensitivity and specificity of diagnostic imaging and makes treatment planning particularly challenging. Without being able to resolve the prostate and bladder in imaging, it becomes far more challenging to determine where to deliver doses. In addition, modern treatment planning software relies on accurate computed tomography (CT)-based imaging to calculate dose deposition. Many MAR techniques have been developed to address this issue [14], and they have helped improve CT and cone-beam computed tomography (CBCT) quality significantly. This is crucial for initial anatomical delineation and contouring, as well as treatment planning and dose calculation. Without high-quality imaging, it is impossible to determine how much of a dose is being delivered and where it is being deposited. Commercial CT MAR solutions are quickly becoming more widespread but are not ubiquitous. At the time of simulation, the CT within our academic department for this case study did not have MAR technologies. Furthermore, the availability of MAR algorithms for CBCT imaging is not nearly as universal. It is, however, highly necessary for cases where precise delineation of the target and healthy tissues is required, especially for oART. Fortunately, HyperSight with MAR was recently released for Varian treatment machines, including the Ethos platform [15]. Imaging with MRI techniques poses similar challenges [16,17], which restricts the ability of MRI-based oART platforms to accurately treat patients with hip arthroplasty.

Given the challenges presented in this case study, it was concluded that most treatment techniques available were not able to deliver a satisfactory plan. The nodal involvement in this case meant that monotherapy brachytherapy would be unable to treat the entire involved region. It was determined that traditional EBRT methods could not sufficiently treat the prostate and nodes while maintaining an acceptable dose to the bowels and rectum. It was decided that the risk of an acute IBD flare-up would make treatment compliance with these modalities too challenging. Proton therapy was investigated as an option because it can deliver a higher-precision dose. However, the interfractional movement of the target and organs at risk (OARs) makes treatment challenging. Because of the complexity and time needed for proton planning, there are currently no online adaptive options using protons [18]. In addition, proton centers lack sufficient on-treatment MAR imaging techniques to accurately set up the patient day-to-day. Finally, MRI-based oART platforms, albeit less accessible and typically requiring longer treatment sessions, would allow for daily oART with good soft tissue contrast and hence volume delineation. However, this technique was contraindicated in this case by the bilateral prostheses. While the titanium implants are MRI-safe, they can heavily distort the magnetic field, leading to large geometric distortions. The patient in this study was evaluated for treatment with each of these modalities and was declined treatment for the reasons listed above. Luckily, the Ethos ART technique, combined with the HyperSight MAR reduction algorithm, can overcome these limitations. Using the MAR algorithm available with HyperSight allows for high-quality image reconstruction to accurately delineate the tumor and healthy tissue positioning. With these high-quality images available, daily oART facilitates a radiation dose distribution optimized for the patient’s anatomy for every treatment. Ultimately, this led to the safe and successful treatment of the patient.

## Case presentation

A 68-year-old male with a medical history of ulcerative colitis and bilateral hip replacement and surgery was diagnosed with very high-risk prostate cancer (Gleason score of 4 + 5 = 9, prostate-specific antigen (PSA) level of 54), classified as cT2ccN1, grade 5. A prostate-specific membrane antigen positron emission tomography (PSMA PET) scan revealed abnormal PSMA activity (standardized uptake value, or SUV 8.8) in the prostate gland and the left obturator lymph node. The patient was prescribed anti-testosterone therapy using Casodex, Lupron, and adjuvant radiotherapy to the prostate gland and the pelvic nodes. The patient also had a 24-year history of ulcerative colitis, which had been well-maintained with medication until flare-ups started occurring in the past few years. He is currently using mesalamine (Rowasa) to manage the ulcerative colitis. In addition, the patient underwent a total hip arthroplasty in 2020, resulting in titanium alloy prostheses in both hips.

ART planning directive

**Figure 1:**
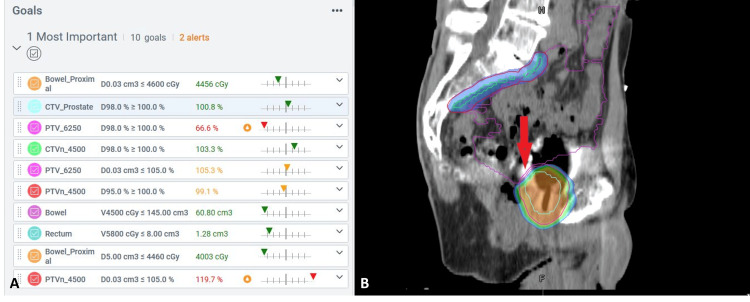
A) Priority 1 goals for the initial phase; B) Reduced PTV coverage due to bowel proximity PTV: Planning target volume; CTV: Clinical target volume

Daily adaptive treatment was prescribed to the prostate (CTV_7000) and pelvic nodes (CTVn_4500), with the targets receiving 6250 cGy and 4500 cGy, respectively, in 25 fractions. In addition, the prostate was prescribed a sequential boost of 750 cGy in three fractions, resulting in a total dose prescribed to the prostate gland of 7000 cGy. The planning target volumes (PTVs) were created using a 7 mm symmetrical expansion margin from the clinical target volume (CTV), except for the PTV_7000, which was expanded 5 mm posteriorly to spare the proximal rectal wall. Smaller expansions were considered for this case, but the decision was made to use the traditional expansions due to the lack of reliable physician coverage for every fraction. The implemented workflow aimed to minimize toxicity with up-to-date contours and optimization, but smaller PTVs will be implemented for future patients as more physicians become involved. Individual bowel loops were contoured daily using artificial intelligence (AI)-generated bowel influencer contours, which were subsequently edited manually to ensure that all bowel loops in the treatment field were accurately segmented. Bowel sparing was prioritized over target coverage in the lower pelvic region adjacent to the prostate, as shown in Figure 1. To help achieve this, a proximal bowel structure was created that captured the bowel within 3 cm of the prostate. Table 1 summarizes the planning directives, including objectives and priorities for the targets and OARs. Beam entrance through the prosthesis was limited using a mean dose constraint on a 3 mm expansion from the bilateral prosthesis. Additional constraints on intermediate and low-dose spread throughout the body-PTV structures were also present but were of lower importance for the intelligent optimization engine (IOE).

**Table 1:** Planning directive PTV: Planning target volume

Targets	Treatment phase	Objective	Priority
PTVn_4500	Ph1	D95% > 100%	1
		D0.03cc < 105%	1
PTV_7000	Ph1	D98% > 100%	1
		D0.03cc < 105%	1
	Ph2	D95% > 100%	Reporting only
		D98% > 100%	1
		D0.03cc < 105%	1
		D95% > 100%	Reporting only
Bladder	Ph1	V5800 < 10%	2
		V4910 < 20%	2
		V4020 < 40%	2
	Ph2	V700 < 10%	2
		V590 < 20%	2
		V480 < 40%	2
Bowel	Ph1	D5cc < 4650 cGy	2
		V4500 < 145cc	1
Bowel proximal to prostate	Ph1	D0.03cc < 4600 cGy	1
		D5cc < 4460 cGy	1
	Ph2	D0.03cc < 540 cGy	1
		D5cc < 540 cGy	1
Rectum	Ph1	V5800 < 8cc	1
		V5800 < 10%	2
		V4910 < 20%	2
		V4020 < 40%	2
	Ph2	V700 < 8cc	2
		V700 < 10%	2
		V590 < 20%	2
		V480 < 40%	2
Penile bulb	Ph1	Dmean < 4500 cGy	4
	Ph2	Dmean < 500 cGy	4
High-Z implant (L&R)	Ph1	Dmean < 893 cGy	2
	Ph2	Dmean < 107 cGy	2

Planning image

**Figure 2:**
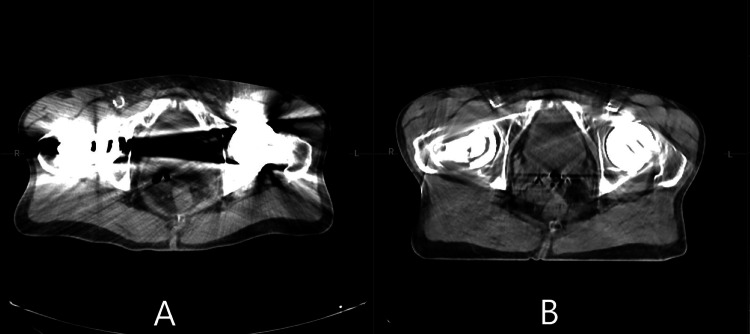
A) Planning CT image; B) HyperSight iCBCT with MAR iCBCT: Image-guided cone-beam computed tomography; MAR: Metal artifact reduction

The initial planning CT image was acquired on a GE RT LightSpeed II scanner without MAR capabilities. At the time of the simulation, Ethos V2, which allows planning on the CBCT image, was not available. However, an imaging-only session was performed on the Ethos system to acquire an image-guided CBCT (iCBCT) with MAR using HyperSight to assist with the segmentation of the target and the OARs due to the significant artifacts present in the planning CT image. The planning CT and iCBCT are shown in Figure 2. The Ethos imaging-only session was not used as a CT simulation in this case, unfortunately. This image was only used to assist in target and OAR delineation by registering it to the CT simulation. Calculation on a CBCT image was not enabled for Ethos at the time of the study. This feature will become available in version 2. The CT simulation, with artifacts, was still used as the reference image, and a daily synthetic image of this was created for daily dose calculation.

Density corrections

The density for the bright-and-dark streaking artifacts caused by the metal implants was overridden with water (1.00 g/cm³) for tissue and titanium alloy (4.42 g/cm³) for the implants. These overrides were deformably propagated to the synthetic CT daily for oART session dose calculation. Validation of the dose calculation accuracy using a simplified density override scheme was verified prior to plan approval by comparing doses calculated with only water and titanium overrides against an identical plan with water, titanium, and bone overrides. Daily OARs and target contours were segmented in the MAR CBCT and then registered to the synthetic planning CT. Water and titanium overrides were also registered to the same synthetic image. The accuracy of the synthetic image was verified by a physicist during the entire course of treatment. There are some inaccuracies introduced by the registration, specifically around water overrides; however, the effect of those on dose calculation is less than 1%. High-density overrides, which could introduce larger errors, are less prone to misregistration as they do not deform with daily anatomy. With dose calculation differences <1% between the calculations, the simplified density mapping was utilized to avoid deformation errors in the density mapping of bone.

Reference plans

The reference plans were created for both treatment phases using 12-field static-beam intensity-modulated radiation therapy (IMRT) plans generated with the IOE. A RapidPlan model was not applied to this planning directive. All OAR constraints were met despite the challenges of treating through metal prostheses. Because achieving the proximal bowel constraint was prioritized over meeting PTV and CTV coverage goals, the dose predicted to be received by 98% of the PTV_7000 was 66.6% in phase 1 and 81% in phase 2. Nonetheless, the predicted dose to the CTV_7000 was greater than 100%. The expectation was that full PTV coverage could be achieved on the treatment days when the bowel was sitting more superiorly relative to the PTV. Meanwhile, the coverage was expected to drop on days when the bowel was proximal to the PTV, and a potential increase in dose hotspots could be expected.

Online treatment sessions

Daily treatment sessions were performed using HyperSight MAR CBCT scans. The prostate, bladder, rectum, and bowel structures were used as influencer structures. All influencers were initially AI-generated and subsequently modified manually by the physicist and physician. The accuracy of the segmentation of the bilateral hip prostheses was verified daily to ensure the avoidance structures were accurate. The treating physician reviewed all CTV contours to ensure they were sufficiently accurate for adaptive treatment. Following online plan selection, a confirmation CBCT was obtained to ensure patient positioning had not changed during the oART workflow.

Data analysis

Two plans are generated for every treatment session: the scheduled plan, which refers to the reference plan calculated on the synthetic CT created from the on-couch session image (CBCT), and the adapted plan, which refers to a plan reoptimized based on on-couch session contours and calculated on the same synthetic CT. Images, structure sets, scheduled and adapted RT plans, and the respective dose distributions were sent to Mobius for second verification. For this study, adaptive and scheduled plan value statistics were queried from Mobius using an internally developed script with Pandas DataFrame methods, along with a Python script provided by Varian in the documentation library, facilitating access to the plan’s raw data in Mobius3D.

Adaptive plan versus scheduled plan performance

**Figure 3:**
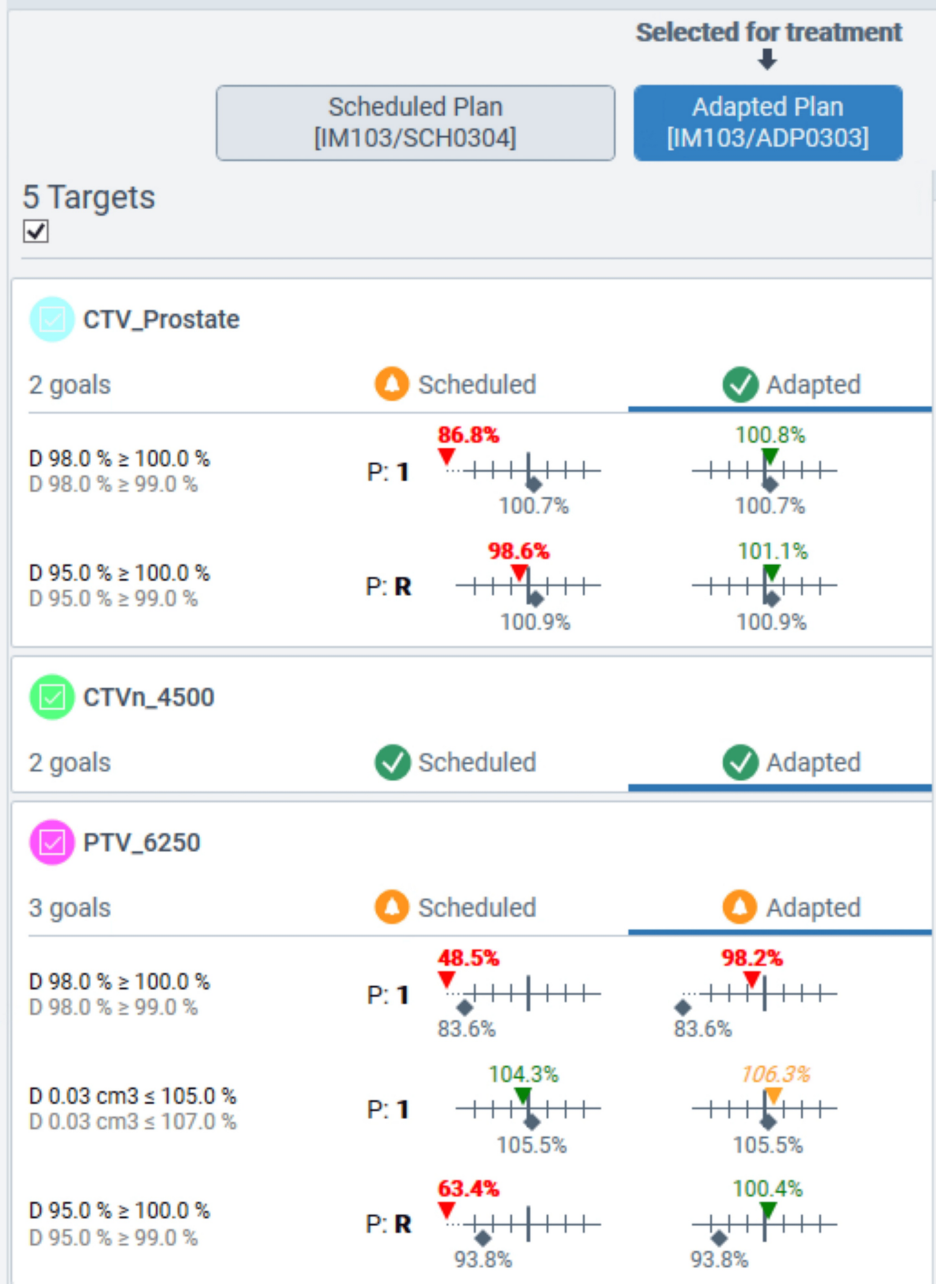
A typical session of the initial phase showing the comparison of the target dose constraints between the scheduled and adapted plans PTV: Planning target volume; CTV: Clinical target volume

**Figure 4:**
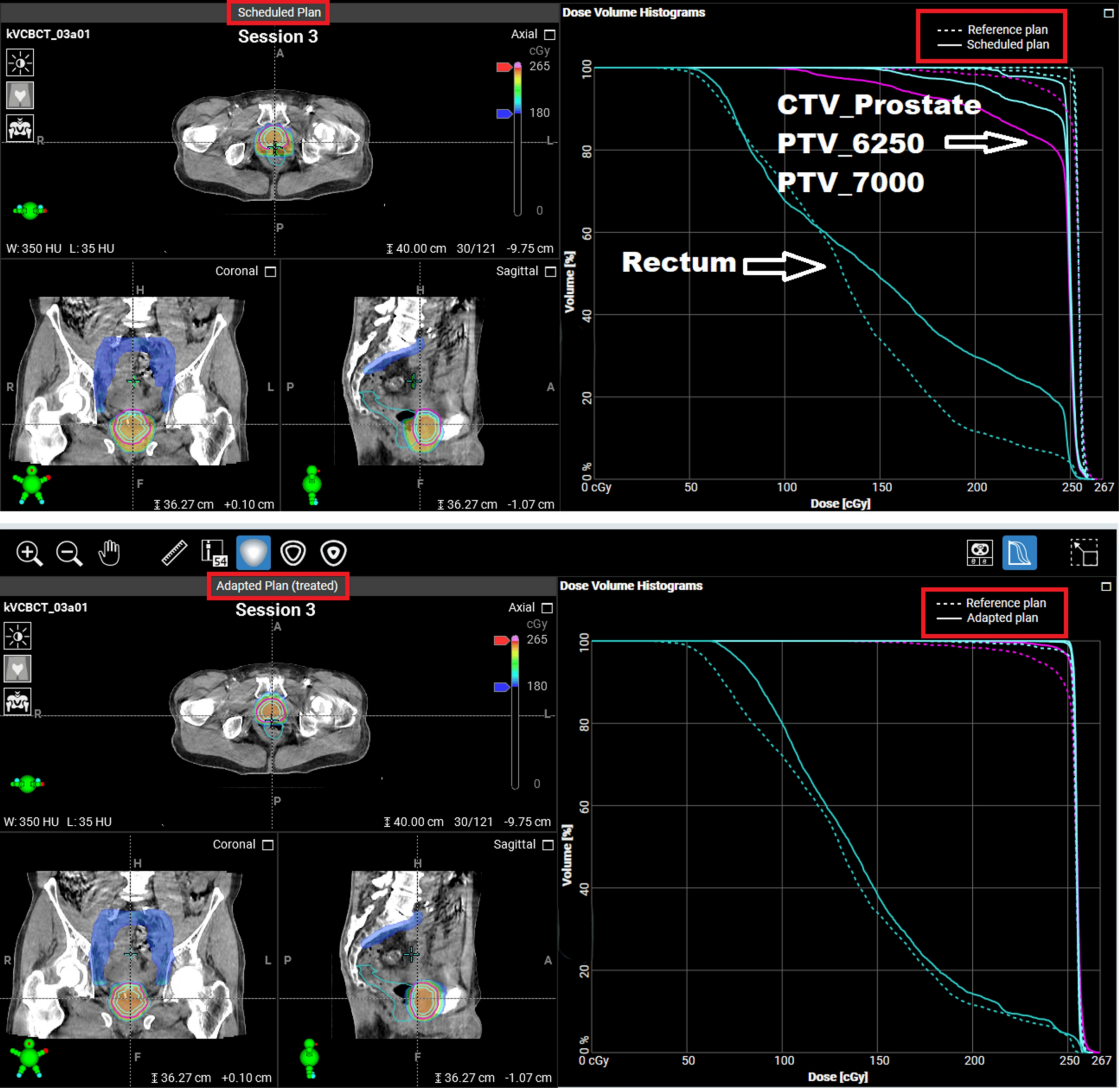
The dose distribution and DVH, comparing the scheduled plan to the reference plan (top) and the adapted plan to the reference plan (bottom), for a typical treatment fraction PTV: Planning target volume; CTV: Clinical target volume; DVH: Dose-volume histogram

**Figure 5:**
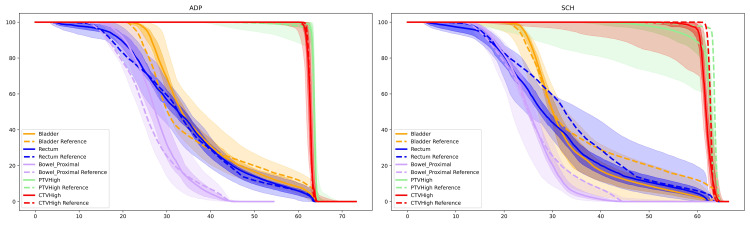
Comparison of the distribution of adaptive plan (left) versus scheduled plan (right) DVHs over the patient’s primary course (6250 Gy in 25 fractions) Dashed lines represent reference plans; the lightly shaded regions are the maximum and minimum extent of the DVH curves; the darkly shaded regions are at the bounds of the 20th-80th percentile of the DVHs; and the solid line is the median DVH curve over all treatments. PTV: Planning target volume; CTV: Clinical target volume; DVH: Dose-volume histogram

All daily treatments were completed within 21 to 41 minutes, with a median time of 29 minutes. Figures 3-4 show the dose comparison between the scheduled and adapted plans of a typical session. As demonstrated in Figures 3-4, because of the changed daily anatomy, target coverage was compromised on the scheduled plan, whereas OARs, such as the rectum or bowel, were overdosed. Figure 5 depicts the dose-volume histogram (DVH) distributions for the two oART plans generated: the adaptive plan and the scheduled plan. Reference DVH values are shown for PTVHigh (PTV_7000), CTVHigh (CTV_Prostate), bladder, rectum, and proximal bowel (Bowel_Proximal). The shaded regions demonstrate that the adaptive plans had a much tighter distribution around the reference plan DVH for all structures while achieving greater values of target coverage for both CTV and PTV structures on average when compared to the scheduled plan. Scheduled plan DVHs demonstrate improved OAR sparing on average but at the expense of target coverage, with certain fractions failing to cover the prostate CTV appropriately. DVH variability was also worse with scheduled plans; notably, the bladder and rectum demonstrated approximately 20% volume variation at 5000 cGy. In contrast, the distribution of OAR DVHs for adaptive plans was much tighter, within 10% over the course of treatment.

**Figure 6:**
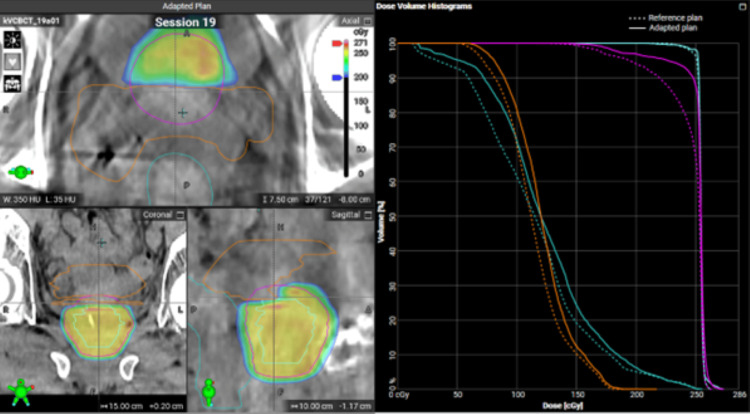
Demonstration of proximal bowel contour relative to PTV_7000 and CTV_Prostate, with the 200 cGy dose wash from the adaptive plan overlaid on the anatomy (left), and corresponding DVH (right) PTV: Planning target volume; CTV: Clinical target volume; DVH: Dose-volume histogram

Adaptive plans achieved the primary objective of the adaptive course of treatment, limiting the maximum point dose to the proximal small bowel to within 5500 cGy for the initial 25 fractions, while maintaining target coverage of the prostate CTV. Figure 6 shows a demonstration of the proximity of the proximal bowel loop to the prostate CTV, highlighting the dosimetric challenges of sparing this section of the bowel. In contrast, the scheduled plan frequently was unable to avoid a full dose to the proximal bowel loop, while also failing to adequately cover the prostate CTV. The adaptive plans demonstrated some indicators of poor plan quality, such as hotspots reaching 115%, but these were within the prostate CTV. Given the geometric challenges of sparing the proximal bowel loop and avoiding beam entrance through the bilateral prostheses, these hotspots were deemed clinically acceptable in some cases.

## Discussion

The use of Ethos with oART and the HyperSight imaging system provided a unique opportunity to treat this particularly challenging patient, which no other modality can fully match at this time [15]. As shown in our case report, the daily adaptive treatment on Ethos was able to deliver a therapeutic dose to the target volumes while sparing the healthy structures to a much greater degree than a non-adaptive treatment would be capable of delivering. This is partially a result of a decrease in the required CTV-to-PTV margin [19,20]. For this patient with ulcerative colitis, this extra sparing - especially to the bowel and rectum - was essential not only to the patient’s comfort and well-being but also to their ability to tolerate and comply with 28 total treatment fractions. An acute flare-up of symptoms might have necessitated a break or complete cessation of treatment. The oART workflow used for this case depended on the clinician’s ability to accurately contour the target and healthy tissue daily. Without this, there would be no way to determine how to proceed with the adaptive treatments. Without the use of the HyperSight MAR abilities, the patient’s bilateral full hip arthroplasty would have made daily imaging infeasible. Instead, we were able to achieve organ delineation that was previously inaccessible in CBCT imaging.

Currently, no other treatment modalities include all the tools necessary to treat this complex patient with the same confidence and quality as the Ethos system. The need for higher-than-usual GI sparing necessitated a treatment technique with the capability for online adaptive treatments. At the time of the patient’s treatment, this narrowed the options down to Varian Ethos and MRI-based linear accelerators (LINACs): MRIdian and Unity. However, this patient was not a candidate for either MR LINAC option because of the presence of his hip prostheses, which would have caused significant image distortion.

Despite the abilities of Ethos oART with HyperSight, there were still some limitations in this case. The lack of MAR technologies on the simulation CT introduced some uncertainty in the initial contouring and planning. The need to deform density overrides created an added challenge with uncertainties, but careful verification of this process kept the inaccuracies to less than 1%. These issues will be significantly lessened for clinics with MAR-capable CTs or Ethos version 2, which will allow for treatment planning based on images taken at the treatment machine.

We are at the forefront of oART, and it is likely that with its continued development, a patient with this level of complexity will be treatable with other modalities in the future. Until then, Varian’s Ethos system with HyperSight MAR methods remains the best viable option.

## Conclusions

Ethos oART with HyperSight provided an effective solution for treating a patient with concurrent prostate cancer, IBD, and bilateral hip arthroplasty. The patient’s case was successfully treated without complications, despite the challenging clinical and technical scenarios.

## References

[1] Peeters ST, Heemsbergen WD, van Putten WL (2005). Acute and late complications after radiotherapy for prostate cancer: results of a multicenter randomized trial comparing 68 Gy to 78 Gy. Int J Radiat Oncol Biol Phys.

[2] Matta R, Chapple CR, Fisch M (2019). Pelvic complications after prostate cancer radiation therapy and their management: an international collaborative narrative review. Eur Urol.

[3] Pinkawa M (2023). Gastrointestinal quality-of-life trajectories after radiotherapy for prostate cancer-which patients suffer from persisting problems?. Cancers (Basel).

[4] Juarez JE, Romero T, Mantz CA (2021). Toxicity after stereotactic body radiation therapy for prostate cancer in patients with inflammatory bowel disease: a multi-institutional matched case-control series. Adv Radiat Oncol.

[5] Feagins LA, Kim J, Chandrakumaran A (2020). Rates of adverse IBD-related outcomes for patients with IBD and concomitant prostate cancer treated with radiation therapy. Inflamm Bowel Dis.

[6] Trotta M, Patel KR, Singh S, Verma V, Ryckman J (2023). Safety of radiation therapy in patients with prostate cancer and inflammatory bowel disease: a systematic review. Pract Radiat Oncol.

[7] Ghilezan M, Yan D, Martinez A (2010). Adaptive radiation therapy for prostate cancer. Semin Radiat Oncol.

[8] Byrne M, Archibald-Heeren B, Hu Y (2022). Varian Ethos online adaptive radiotherapy for prostate cancer: early results of contouring accuracy, treatment plan quality, and treatment time. J Appl Clin Med Phys.

[9] Byrne M, Teh AY, Archibald-Heeren B (2024). Intrafraction motion and margin assessment for Ethos online adaptive radiotherapy treatments of the prostate and seminal vesicles. Adv Radiat Oncol.

[10] McPartlin AJ, Li XA, Kershaw LE (2016). MRI-guided prostate adaptive radiotherapy - a systematic review. Radiother Oncol.

[11] Pathmanathan AU, van As NJ, Kerkmeijer LG (2018). Magnetic resonance imaging-guided adaptive radiation therapy: a “game changer” for prostate treatment?. Int J Radiat Oncol Biol Phys.

[12] Müller GM, Lundin B, von Schewelov T, Müller MF, Ekberg O, Månsson S (2015). Evaluation of metal artifacts in clinical MR images of patients with total hip arthroplasty using different metal artifact-reducing sequences. Skeletal Radiol.

[13] Boas FE, Fleischmann D (2012). CT artifacts: causes and reduction techniques. Imaging Med.

[14] Selles M, van Osch JA, Maas M, Boomsma MF, Wellenberg RH (2024). Advances in metal artifact reduction in CT images: a review of traditional and novel metal artifact reduction techniques. Eur J Radiol.

[15] Robar JL, Cherpak A, MacDonald RL (2024). Novel technology allowing cone beam computed tomography in 6 seconds: a patient study of comparative image quality. Pract Radiat Oncol.

[16] Peschke E, Ulloa P, Jansen O, Hoevener JB (2021). Metallic implants in MRI - hazards and imaging artifacts. Rofo.

[17] Jungmann PM, Agten CA, Pfirrmann CW, Sutter R (2017). Advances in MRI around metal. J Magn Reson Imaging.

[18] Albertini F, Matter M, Nenoff L, Zhang Y, Lomax A (2020). Online daily adaptive proton therapy. Br J Radiol.

[19] Christiansen RL, Dysager L, Hansen CR (2022). Online adaptive radiotherapy potentially reduces toxicity for high-risk prostate cancer treatment. Radiother Oncol.

[20] Morgan HE, Wang K, Yan Y (2023). Preliminary evaluation of PTV margins for online adaptive radiation therapy of the prostatic fossa. Pract Radiat Oncol.

